# Student perception of two different simulation techniques in oral and maxillofacial surgery undergraduate training

**DOI:** 10.1186/1472-6920-11-82

**Published:** 2011-10-12

**Authors:** Bodil Lund, Uno Fors, Ronny Sejersen, Eva-Lotta Sallnäs, Annika Rosén

**Affiliations:** 1 Division of Oral and Maxillofacial Surgery, Department of Dental Medicine, Karolinska Institutet, Stockholm, Sweden; 2 Department of Computer and Systems Sciences, Stockholm University, Sweden and Department of Learning, Informatics, Management and Ethics, Karolinska Institutet, Sweden; 3 Department of Learning, Informatics, Management and Ethics, Karolinska Institutet, Sweden; 4 School of Computer Science and Communication, KTH, Stockholm, Sweden

## Abstract

**Background:**

Yearly surveys among the undergraduate students in oral and maxillofacial surgery at Karolinska Institutet have conveyed a wish for increased clinical training, and in particular, in surgical removal of mandibular third molars. Due to lack of resources, this kind of clinical supervision has so far not been possible to implement. One possible solution to this problem might be to introduce simulation into the curriculum. The purpose of this study was to investigate undergraduate students' perception of two different simulation methods for practicing clinical reasoning skills and technical skills in oral and maxillofacial surgery.

**Methods:**

Forty-seven students participating in the oral and maxillofacial surgery course at Karolinska Institutet during their final year were included. Three different oral surgery patient cases were created in a Virtual Patient (VP) Simulation system (Web-SP) and used for training clinical reasoning. A mandibular third molar surgery simulator with tactile feedback, providing hands on training in the bone removal and tooth sectioning in third molar surgery, was also tested. A seminar was performed using the combination of these two simulators where students' perception of the two different simulation methods was assessed by means of a questionnaire.

**Results:**

The response rate was 91.5% (43/47). The students were positive to the VP cases, although they rated their possible improvement of clinical reasoning skills as moderate. The students' perception of improved technical skills after training in the mandibular third molar surgery simulator was rated high. The majority of the students agreed that both simulation techniques should be included in the curriculum and strongly agreed that it was a good idea to use the two simulators in concert. The importance of feedback from the senior experts during simulator training was emphasised.

**Conclusions:**

The two tested simulation methods were well accepted and most students agreed that the future curriculum would benefit from permanent inclusion of these exercises, especially when used in combination. The results also stress the importance of teaching technical skills and clinical reasoning in concert.

## Background

Interactive teaching has been shown to improve students' achievements in oral and maxillofacial undergraduate training [1]. Due to constant and increasing development within the field of medicine and dentistry the clinician needs to develop effective tools for life-long learning. Although curriculum design does not seem to influence the attitude towards life-long learning [2] an interactive teaching modality provides the student with the necessary skills to independently train to solve clinical problems and thereby meet requirements of the future [3].

In surgery, the introduction of simulators has provided novel possibilities for a student oriented environment to develop practical skills. However, the success of a certain simulator is dependent on its introduction to a well designed curriculum and that its contribution to learning and advantages over traditional teaching has been well assessed [4]. This in turn requires close cooperation between clinical tutors and representatives of the industry during the development and implementation of a surgery simulator. An interesting simulator is the Forsslundsystem AB Oral Surgery Simulator http://www.forsslundsystems.se/ that uses a haptic technology which enables the kinaesthetic and tactile sensation of shape, texture and friction of the operating field. Consequently the surgeon in training may receive real-time knowledge about the surgical procedure based on the modalities vision, hearing and touch [5].

Mandibular third molar surgery, as any kind of surgery, besides demanding practical skills also requires good clinical reasoning skills to make the correct diagnosis and for treatment planning (including ability to determine the difficulty of the planned operation and if there is a need to refer the case to a specialist or not). The use of virtual patients has been suggested to be an important teaching aid for development of such clinical reasoning [6]. The Virtual Patient (VP) Simulation system Web-SP is an interesting tool for training these kinds of clinical reasoning skills and has been shown to give high acceptance among medical and dental students [7,8]. It has also been shown that practicing with virtual patients in Web-SP gives a higher retention compared to traditional learning methods [9]. Web-SP is a software which provides a common generic platform facilitating for teachers in health care sciences to create interactive patient cases without the dependence on computer specialists [10].

The undergraduate curriculum in oral and maxillofacial surgery for students in their final (5th) year at the Department of dental medicine, Karolinska Institutet, corresponds to three weeks full time studies. Previously the main part of this course basically contained lectures and observational teaching. Besides problems such as economical cut-down, increased number of students, reduction of teacher resources, this old curriculum faced major problems such as making the students passive. Furthermore the unaltered patient-flow caused uneven learning conditions and was very stressful for the teachers and assisting staff. A new curriculum was developed where the bulk of observational education was replaced by interactive seminars, improving the course [11].

A constant desire, apparent from the yearly student surveys, has been practical training in surgical removal of mandibular third molars. Due to lack of resources, this kind of clinical supervision has so far not been possible to implement. Likewise, the Swedish Dental Association has conducted several studies on the graduated dentists' perception of their dental education. A clear, dominating and recurring apprehension was that the amount of practical training in undergraduate oral and maxillofacial surgery training was insufficient [12].

In an attempt to further improve the curriculum, and partly meet the expectations of the students in terms of increased practical moments, simulator aided teaching has been proposed as a way to improve both clinical reasoning skills as well as practical surgery skills. The purpose of this study was to investigate undergraduate students' perception of two different simulation methods for oral and maxillofacial surgery.

## Methods

### Participants

All undergraduate students (n = 54), participating in the oral and maxillofacial course during their final year of dental education at the Department of dental medicine, Karolinska Institutet, Stockholm, Sweden, were offered to participate in the study. Forty-seven of these students agreed to be included in the study and signed a written informed consent. Two stations with the Forsslund systems third molar surgery simulator as well as five stations with the Web-SP system was set-up. The teaching staff resources consisted of three persons trained in oral and maxillofacial surgery, one assisted at each oral surgery simulator and the third aided the students working with Web-SP. Two technicians were also available to solve any problem or question of technical nature regarding the oral surgery simulator. An ethical approval was obtained from the local ethics committee at the Karolinska University Hospital, Huddinge, prior to on-set of the study.

### Web-based virtual simulation of patients (Web-SP) stations

The aim with Web-SP is to mimic the live patient situation, where medical history and data from physical examination is collected by the student, who subsequently diagnose the patient and suggests therapy. Thus, the student is not passively provided with the necessary data but has to acquire it by asking relevant questions, suggest correct physical examinations and laboratory/imaging tests. The system provides an optional built in feed-back at the end of each exercise [13]. In the current study the feed-back was performed by a teacher aided group discussion at the end of the session. Based on data from true cases, three different oral surgery patient cases were created, where one or more mandibular third molar were to be surgically removed. The cases were designed using the predefined case template available in the Web-SP, as previously described [9]. The cases were created by one of the teachers at the oral and maxillofacial surgery department.

### Oral Surgery Simulator stations

Two equal sets of a mandibular third molar surgery simulator prototype of the Oral Surgery Simulator (Forsslund Systems AB, Stockholm, Sweden) were used in the study. The simulator provides hands on training of bone removal and tooth sectioning in third molar surgery through a haptic devise. The technology of haptic feed-back systems gives a realistic sensation of drilling concerning anatomy, vibration and resistance of the different tissues i.e.alveolar bone, enamel, dentin and pulpal tissue. The simulated procedure had been created from CT scans of an authentic case of a mesioangulated mandibular third molar. The tactile sensation has been fine-tuned by feed-back from trained oral and maxillofacial surgeons testing the simulator.

### Implementation of simulators

The seminar lasted for 3 hours and was divided in three parts: an oral introduction, exercises in both the Oral Surgery Simulator and in the Web-SP, respectively, followed by a group discussion.

In the oral introduction the students were given a short presentation of the Web-SP system, the Oral Surgery Simulator and the aim of the seminar. Thereafter a white-board aided step-by-step description of the practical steps of surgical mandibular third molar removal was given.

In the simulation part of the seminar the students rotated between the two different simulation stations as follows:

1. The oral surgery simulator 

Each student participating in the study was given 15 minutes of hands on training in the Oral Surgery Simulator. During the session a teacher trained in oral and maxillofacial surgery was present introducing the student to the task and giving verbal feed-back. Staff, well familiar with the simulator, were standing by, ready to provide technical assistance if needed.The students also had the opportunity to observe a fellow student work in the simulator.

2. Web-SP

 In the exercise with the Web-SP Virtual patient simulation, the students worked independently in pairs with the cases for approximately 60 minutes, during which a teacher was available to answer possible questions.

The seminar ended with a group discussion where the three patient cases in Web-SP were examined and possible questions regarding mandibular third molar removal in general were clarified.

### Assessment of student perception

After the seminar the students were asked to fill in an anonymous questionnaire consisting of different statements regarding the two different simulation techniques. The students were asked to rank their answers according to a 6-graded scale, where 1 meant "totally disagree" and 6 "agree completely". There were three categories of questions or statements: those of demographic and general issues; regarding Web-SP; and finally regarding the oral surgery simulator (Table 1). The issues of the general questions were besides gender and age, the extent of computer usage and previous experience of computer aided education. The simulation related questions inquired if the students believed that the exercises had resulted in improved skills and/or new knowledge, if simulation should be included in future courses, and to which extent the oral surgery simulator was experienced as realistic. Space was provided for free comments in the last part of the questionnaire.

**Table 1:** Issues Assessed in the Questionnaire

**A. Demographic and general questions**
1. Gender
2. Age
3. I use computer and/or computer games at home.
4. I have previous experience of computer aided education.
**B. Web-SP statements**
5. The patient cases in Web-SP gave me more knowledge about the difficulties with judging whether a mandibular third molar should be removed or not.
6. Web-SP improved my knowledge regarding diagnostics in oral surgery cases.
7. Web-SP gave me more knowledge whether a case should be referred or not.
8. Web-SP should be included in the course in the future.
9. I would like to have more Web-SP cases to work with.
**C. Oral surgery simulator statements**
10. The simulator reproduced mandibular third molar surgery in a realistic way.
11. I have improved my skills in mandibular third molar surgery after the practise with the oral surgery simulator.
12. The practise in the oral surgery simulator has given me new knowledge.
13. The work in the oral surgery simulator felt realistic.
14. Practise in the simulator should be included in the course in the future.
15. I would like more training with the simulator.
**D. Both Web-SP and simulator statement**
16. It was a good idea to use the oral surgery simulator and Web-SP on the same teaching occasion.
**E. Open questions**
17. What was the best part of the course?
18. What was the worst part of the course?

## Results

Filled out questionnaires were received from 43 participants corresponding to a 91.5% (43/47) response rate. Two of these were not complete, resulting in answers from only 41 individuals for questions 10 to 18. The age of the participants ranged from 23 to 45 with a mean value of 26.2 years. The demographic data showed a gender distribution of 29 females and 12 males. One participant did not state sex affiliation. The range of domestic usage of computers and computer games ranged from "totally disagree" (score 1; n = 4) to "agree completely" (score 6; n = 8) with a mean value of 3.9. Previous experience of computer aided education was moderate with a mean score value of 3.5, where 2 individuals stated "totally disagree" and 1 "agree completely".

**Figure 1:**
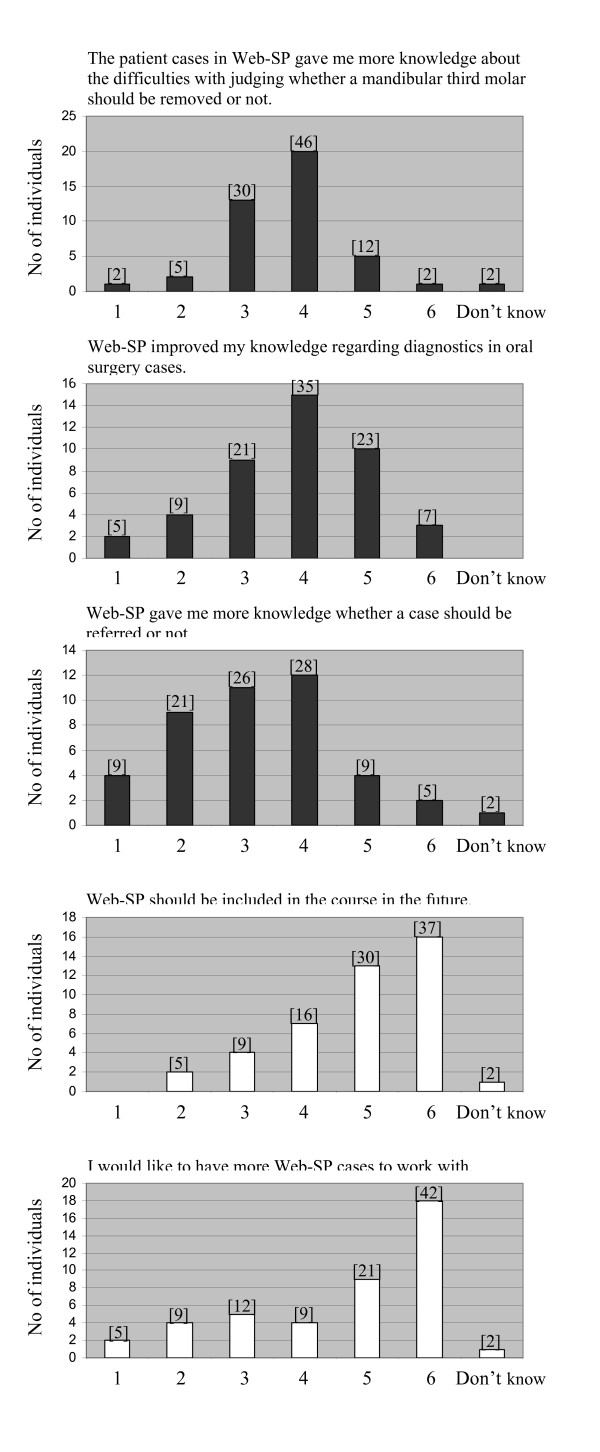
**Diagrams illustrating distribution of participants' scoring of various statements, regarding Web-SP, where 1 is analogous to "totally disagree" and 6 to "agree completely"**. The statement in question is designated on top of each diagram. Numbers in brackets denotes the bars' corresponding percentage of total number of answers.

The students reported that they were positive to Web-SP and that it should be included in the future course (mean 4.9), as well as a desire to have more cases to work with in the future (mean value 4.6), see Figure 1. However, although the aim of the curriculum was to improve students' clinical reasoning skills regarding therapeutic decisions, diagnostics and judgement of whether a case should be referred to specialist or not, the students rated their possible learning outcomes lower than anticipated (mean value 3.7, 3.8 and 3.2 respectively). In free text comments regarding Web-SP, the importance of feedback and teacher assisted discussion was emphasized (n = 3). Three individuals stressed that they felt it was time-consuming to obtain/find the relevant patient data when working with the cases in Web-SP (too much clicking).

**Figure 2:**
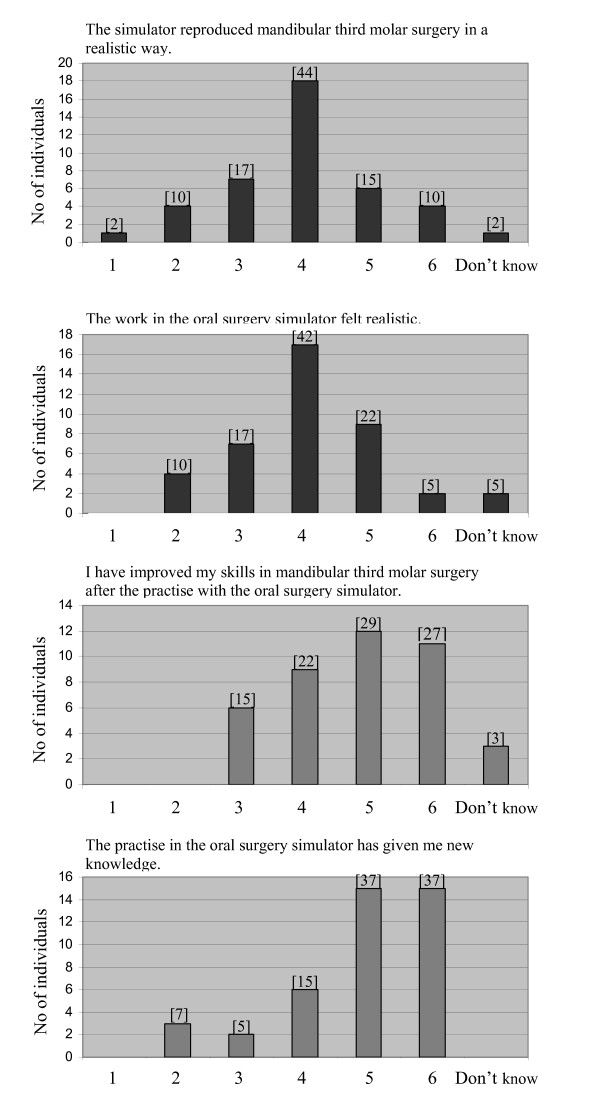
**Diagrams showing participants' scoring of different statements, regarding the oral surgery simulator, where 1 is analogous to "totally disagree" and 6 to "agree completely"**. The statement in question is designated on top of each diagram. Numbers in brackets denotes the bars corresponding percentage of total number of answers.

**Figure 3:**
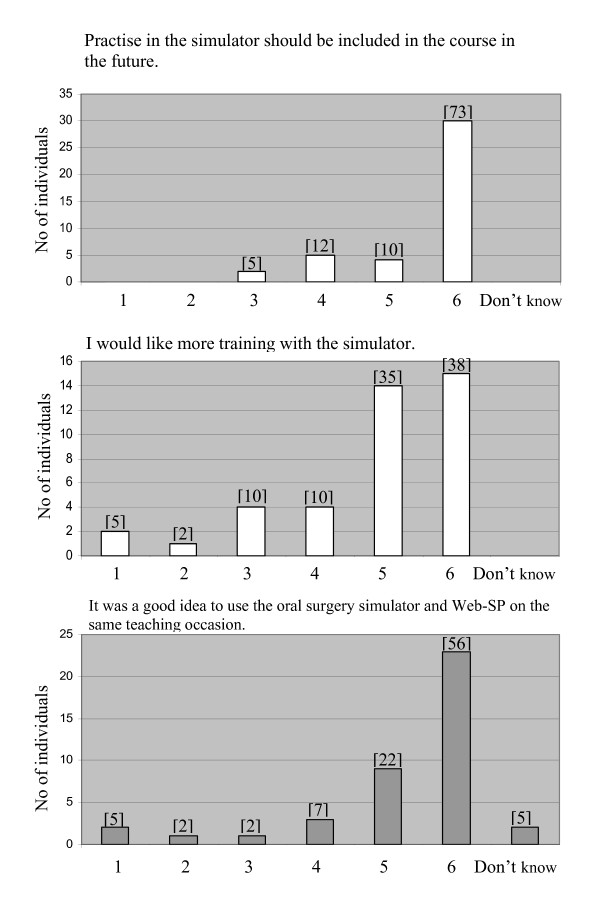
**Figure illustrating distribution of participants' scoring of statements, regarding the oral surgery simulator, where 1 is analogous to "totally disagree" and 6 to "agree completely"**. The corresponding statement is shown above each diagram. Numbers in brackets over each bar is the percentage of total number of answers.

The ratings of whether the simulator reproduced a third molar surgery procedure in a realistic way, and if the work in the oral surgery simulator felt realistic, were on average 3.9. The students' perception of improved skills and acquisition of new knowledge after the simulator exercise was scored high to an average value of 4.7 and 4.9, respectively. For details see Figure 2. The majority of the students agreed that the simulator should be included in the course and wished to have more training in the simulator, average scoring of 5.5 and 4.8, respectively (Figure 3). A total of 56% of the students strongly agreed that it was a good idea to use the simulator and Web-SP on the same teaching occasion (Figure 3). In the free comments section several students stated that they found the exercises in the simulator fruitful and entertaining (n = 10). Different suggestions, mainly graphic, how to improve the simulator (n = 9) was also reported. Some individuals wished to have more cases to work with (n = 3). 

There was no apparent correlation between the attitude that the simulation methods should be included in the future course and gender, computer experience and accustomedness with computer aided teaching.

## Discussion

The results of the present study show that the attitude towards the simulator techniques tested, in terms of agreement that the simulator exercises should be included in the future curriculum, was high. This is in agreement with another report showing that dentistry students preferred e-Learning tools to higher-order learning activities [13]. The acceptance of the simulator techniques could not be correlated to previous usage of computers or previous experience of computer aided teaching, which might indicate that both simulation methods are user-friendly and not dependent on previous experience beyond what could be expected from dental students.

The aim of the Web-SP activities was to improve students learning in therapeutic decisions, diagnostics and judgement of whether a case should be referred or not. These issues were rated slightly positive, but lower than anticipated (reflected in questions 5 through 7) by the students. One explanation for this rather low figure is probably the limited number of available patient cases since the desire to have more cases to work with was given a high score as well as the suggestion of including this activity in the future curriculum. It also seems that the acceptance of Web-SP was dependent on the feed-back given in the teacher aided discussion. This is supported by a previous study on factors influencing dental students' usage of the Web-SP, where students favoured patient cases with feed-back compared to those lacking feed-back [8]. Objectives to use simulator technologies may range from improvement of learning conditions to cost-effective teaching. Since the number of dental educators continues to decrease, educational technologies can offer important means to reduce teaching time [14]. This, however, is dependent on the amount and type of feed-back, which these technologies require. It is therefore important to clarify how different type of feed-back, e.g. verbal by dental teacher, assistant university teacher or fellow student, affects learning in the simulators. The issue of feed-back is also very important when implementing new teaching methods and should therefore be addressed in future studies regarding the oral surgery simulator.

A few individuals (n = 3) criticized Web-SP for requiring extensive clicking when working with the patient cases. This was an unexpected view and should perhaps warrant a more thorough introduction of the Virtual Patient system to the students, since it reflects a misunderstanding of the Web-SP exercise. The purpose is not to click on every possible option in the program but to train/practice to find the relevant questions/laboratory tests/x-ray examination for the individual case as in a true clinical situation.

Although the perception of realistic reproduction of mandibular third molar surgery in the oral surgery simulator was rated as just above average, the students still perceived the exercise in the simulator fruitful and developing, which indicates that an oral surgery simulator may play an important role in oral and maxillofacial undergraduate training. The importance of feed-back from senior teachers, during the practice in the simulator, was emphasised by the students in the free comments. This warrants for further studies regarding the optimal nature of the concomitant tutoring while working in the simulator.

A key issue in successful surgery is the combination of clinical reasoning skills and technical skills. The combination of the oral surgery simulator and Web-SP gives the students opportunity to practice these skills in concert, which is of outmost importance for training discerning clinicians. It is therefore gratifying that most students agreed that these teaching activities should be used on the same teaching occasion, which indicates a judicious approach to surgery. A future improvement of the teaching session could therefore be, besides increasing the number of patient cases, to have the same cases in Web-SP as in the oral surgery simulator, to further emphasise the coupling of these two skills.

## Conclusions

The two tested simulation methods were well accepted and most students agreed that the future curriculum would benefit from permanent inclusion of these exercises, especially when used in combination. To fulfil the aims of the seminar, to improve clinical reasoning and technical skills, the students need to be exposed to more virtual patient cases. Furthermore, feedback from teachers during the seminar was considered important. Previous experience of computers and computer aided teaching did not seem to affect students' perception of the simulators.

## Competing interests

The authors declare that they have no competing interests.

## Authors' contributions

BL and AR conceived the study, organized and designed the teaching session and contributed with experience of traditional teaching in oral and maxillofacial surgery undergraduate training. UF contributed which experience of Web-SP and was active in the planning and outline of the study design as well as interpretation of obtained data. RS has provided technical support and experience regarding implementation of Web-SP in undergraduate training. ES has provided with knowledge and experience regarding behavioural science and evaluation of simulators with haptic devices. The manuscript was read and approved by all authors.

## Pre-publication history

The pre-publication history for this paper can be accessed here:

http://www.biomedcentral.com/1472-6920/11/82/prepub
